# Adult Female with Abdominal Pain

**DOI:** 10.5811/westjem.2015.10.28538

**Published:** 2015-12-01

**Authors:** Sarah E. Frasure, Kimberly Stanford

**Affiliations:** Brigham and Women’s Hospital, Department of Emergency Medicine, Boston, Massachusetts

## CLINICAL SCENARIO

A 42-year-old female presented to the emergency department with diffuse abdominal pain, vaginal discharge, and a fever of 102°F. She described multiple recent male sexual partners, with inconsistent condom use. Her vital signs were unremarkable. Her physical exam was notable for moderate right lower quadrant tenderness to palpation. There was no cervical motion tenderness. The emergency physician performed a bedside abdominal ultrasound (Video), and subsequently ordered a computed tomography (Figure), which confirmed the diagnosis.

## DIAGNOSIS

Tubo-Ovarian Abscess (TOA). The patient was admitted to the gynecology service and provided with intravenous antibiotics. Approximately 70cc of purulent material was drained from the abscess. Of note, chlamydia and gonorrhea cultures were both negative. She was discharged from the hospital with a two-week course of oral antibiotics. A TOA is the most severe manifestation of pelvic inflammatory disease (PID), which is traditionally defined as an infection of the upper genital tract. TOAs are most commonly caused by *Chlamydia trachomatis* and *Neisseria gonorrhoeae*.^1^ Although PID is a clinical diagnosis, abdominal imaging can identify potentially life-threatening complications, such as pyosalpinx or TOA. Classic ultrasound findings of TOA include a complex, mixed solid and cystic mass in the pelvis, which is often preceded by a dilated, fluid-filled, tubular structure in the adnexae characteristic of hydro/pyosalpinx.^2^ These findings were quickly recognized on bedside ultrasound, facilitating the prompt diagnosis of TOA by an emergency physician.

## Figures and Tables

**Figure:**
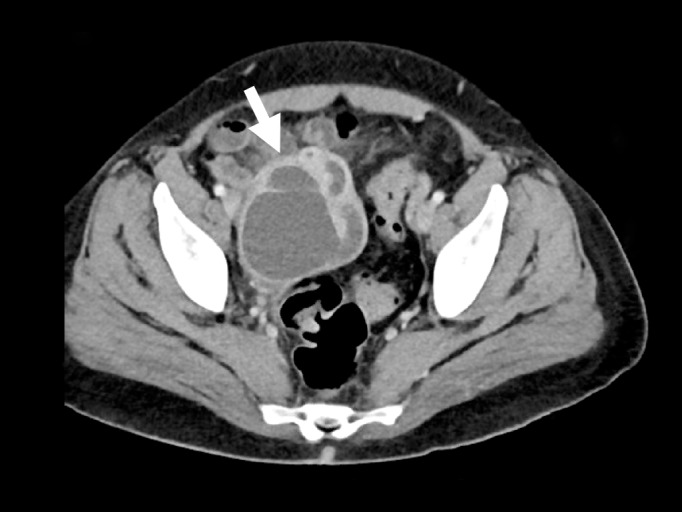
The computed tomography finding which confirmed the diagnosis.

**Video:** A trans-abdominal video sweeping across the pelvis.
